# Damage Localization, Identification and Evolution Studies during Quasi-Static Indentation of CFRP Composite Using Acoustic Emission

**DOI:** 10.3390/polym15244633

**Published:** 2023-12-07

**Authors:** Jinbo Du, Han Wang, Liang Cheng, Yunbo Bi, Di Yang

**Affiliations:** 1 State Key Laboratory of Fluid Power and Mechatronic System, School of Mechanical Engineering, Zhejiang University, Hangzhou 310027, China; 11925018@zju.edu.cn (J.D.); wang_h2018@zju.edu.cn (H.W.); chengliang@zju.edu.cn (L.C.);; 2 Key Laboratory of Advanced Manufacturing Technology of Zhejiang Province, School of Mechanical Engineering, Zhejiang University, Hangzhou 310027, China

**Keywords:** CFRP, acoustic emission, continuous wavelet transform, damage evolution

## Abstract

Quasi-static indentation (QSI) experiments are conducted to investigate the localization, identification and evolution of induced damage in laminate composite up to delamination initiation using acoustic emission (AE) techniques. In this study, we propose a continuous wavelet transform (CWT)-based damage localization method for composites, which can simultaneously identify two damage modes, namely matrix cracking and delamination. The experimental findings demonstrate that the proposed algorithm, which utilizes the arrival time difference within a specific frequency band of the AE signal, effectively reduces the average location error from 3.81% to 2.31% compared to the existing method. Furthermore, the average signal location time has significantly decreased from several minutes to a mere 2 s. Matrix cracking and delamination are identified based on the maximum frequency band of CWT. Both types of damage exhibit prominent peaks within the 40 kHz–50 kHz frequency range, indicating their shared nature as manifestations of matrix damage, albeit with distinct modes of presentation. The first damage pattern that occurs is matrix cracking, succeeded by delamination damage. The nonlinear phase of the mechanical response curve is associated with the rapid aggregation of matrix cracking. Before the onset of macroscopic delamination damage, microscopic delamination damage begins to accumulate. A concentration of high-energy delamination damage signals predicts the initiation of macroscopic delamination.

## 1. Introduction

The utilization of carbon-fiber-reinforced polymers (CFRP) is experiencing rapid growth in various industrial sectors, including aerospace, aeronautics, defense and building constructions [1,2]. Composite materials can offer numerous advantages, primarily owing to their exceptional mechanical strength and stiffness in relation to their weight. However, the complex composition of composite materials makes it hard to distinguish different damage pattern under external loads, such as the common quasi-static indentation load and low-velocity impact [3,4]. These kinds of load do not leave obvious damage on the surface of the composite structure, but there is already significant matrix cracking and delamination inside [5], which seriously affect the load-bearing performance and service life of structure. Therefore, clarifying the location of internal damage and the stage of damage development is of great significance to ensure the normal service of composite structures.

Accurate damage localization and identification are essential for assessing the remaining performance of composite structures. Several non-destructive testing methods, including ultrasonic C-scan [6,7], thermographic studies [8,9] and X-rays [10,11], have been employed to assess the severity of the damage within composite materials. These methods are effective in identifying hidden damage that cannot be seen with the naked eye. However, they are incapable of providing a real-time assessment of the structure’s condition at the usage site. The timely detection and localization of damages are crucial to maintain the load-bearing performance of composite structures.

Acoustic emission (AE) technology is a promising non-destructive testing method that detects the acoustic signals emitted by small strains or damage within materials to evaluate the damage situation [12,13]. There have been significant advancements in the research of source localization based on AE signals. One notable method is the delta T mapping location method, introduced by Baxter et al. [14]. Other researchers have investigated similar source location methods used in anisotropic composite materials [15,16] and achieved good localization results. However, most of these methods primarily rely on the first given threshold crossing as the arrival time for the AE source event. The propagation wave of an AE pulse is dispersive due to the anisotropic nature of composite materials. Even slight variations in the shape of AE signal can significantly impact the accurate determination of the signal’s arrival time. Aljets et al. [17] have developed a source location method using a triangular sensor array based on an AE source. In their method, they consider the signal arrival time and the time difference between the two wave modes as calculation parameters. Mostafapour et al. [18] have proposed an algorithm that utilized wavelet transform (WT) and cross-time frequency spectrum that decomposes the AE signal into a frequency band ranging from 0.125 to 0.25 MHz in order to calculate the time delay at which the cross-time frequency spectrum reaches its maximum value. Jeong et al. [19] have proposed a method to calculate the group velocity using the Gabor wavelet transform. They consider the group velocity to be a function of frequency. The results indicate that the peak amplitude of the wavelet transform corresponds to the signal’s arrival time in a specific frequency band. Sikdar et al. [20] have used the continuous wavelet transformation (CWT) of AE signals as a training set to train convolution neural networks (CNN) and achieved good localization results in the test set.

Damage classification in composite materials based on time-domain [21] and frequency-domain [22] parameters have been comprehensively investigated. In the time domain, commonly used parameters, such as amplitude, duration, rise time and count, are threshold-related parameters and do not fully characterize the damage event. On the other hand, frequency-domain parameters like peak frequency and centroid frequency are employed for damage mode classification. However, Maillet [23] pointed out that taking peak frequency alone for damage identification may lead to errors due to the sensitivity of the signal to variations in the propagation path. To address this limitation, the WT has been utilized, as it provides both the time and frequency information of the signal. WT is a more intuitive method for decomposing time sequence data, allowing simultaneous time-frequency localization. Transforming the signal into the time-frequency domain enables more precise feature extraction. K Mohamed et al. [24] conducted tensile tests on adhesively bonded single-lap joints and utilized CWT to identify different failure modes based on frequency band. They identified matrix failure, fiber-matrix debonding and fiber failure by analyzing the high-energy area in the time-frequency domain. Similarly, Karimi et al. [25] studied the characteristic of AE signals released during the drilling process. They found that the frequency components of the wavelet packet decomposition at each stage were different, indicating various damage modes, such as matrix cracking and fiber breakage. While the above works made significant contributions to the source location and damage identification of CFRP, it is worth noting that existing methods mostly separate the localization and damage classification.

At present, to the best of the authors’ knowledge, there is still a lack of studies addressing the localization of specific damage modes and the analysis of damage evolution under QSI loading. At the same time, existing localization methods are not accurate enough and are time consuming. Therefore, the main objective of this research is to accurately localize and identify damage to laminate composites using AE techniques to investigate the evolution of induced damage during the initial stage QSI. To achieve this end, a wavelet transform-based source localization method is first introduced to detect and localize the damage signal. The accuracy of the proposed algorithm is verified through PLB tests. Two damage modes, namely matrix cracking and delamination, are identified based on the main frequency component of the AE signal CWT. C-scan results after QSI illustrate that the localization method can accurately locate internal damage. Based on the distribution of damage events, the evolution of two types of damage matrix cracking and delamination is analyzed. The localization and classification results can be used to explain the evolution pattern of matrix cracking and delamination damage under early-stage QSI loading.

## 2. Experimental Procedure

### 2.1. Material Manufacture

The composite plate used in this study was fabricated using USN25000 (T700/7901) prepreg, which was supplied by Weihai Guangwei Composites Co., Ltd. (Weihai, China). The fiber volume fraction of the composite plate was measured to be 64%, and it had a thickness of approximately 0.25 mm. The composite plate was cut into a size of 330 mm × 330 mm and 150 mm × 100 mm with a quasi-isotropic stacking sequence of [45/0/–45/90]_2s_, which represented a composite laminate with four layers oriented at 45 degrees, 0 degrees, −45 degrees and 90 degrees, repeated twice, and the laminate was symmetric. For the purpose of evaluating the proposed location algorithm, a test region measuring 200 mm × 200 mm was selected on the composite surface. The test region was further divided into smaller squares with a side length of 10 mm, resulting in a total of 441 grid nodes.

### 2.2. PLB Test and AE Setup

**Figure 1:**
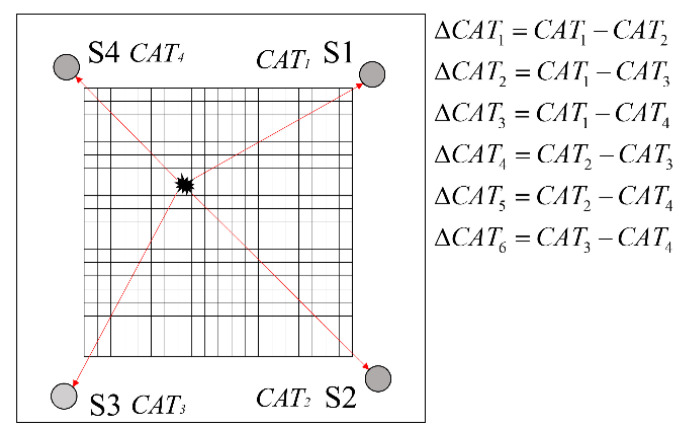
Illustration for the AE sensors placement.

**Figure 2:**
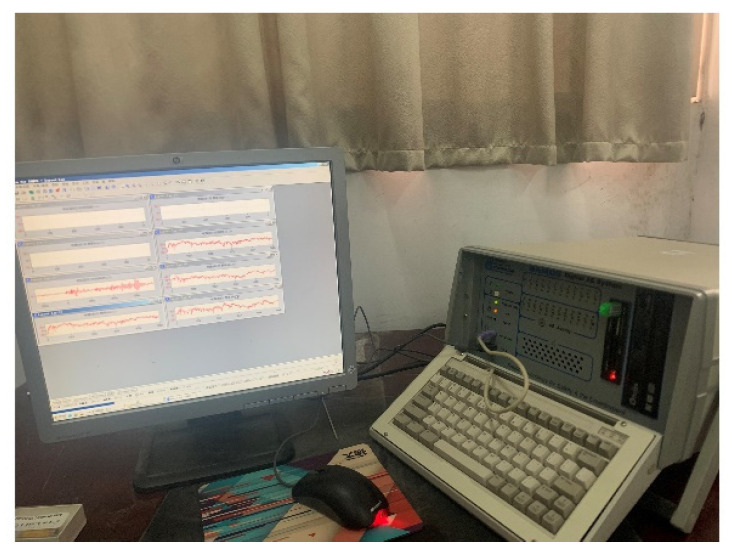
AE device.

The experimental findings have demonstrated the occurrence of acoustic emission phenomena during material fracture. The pencil lead break (PLB) method is commonly employed as a dependable acoustic source for conducting laboratory experiments aimed at simulating the AE source events resulting from material damage. The PLB method involves breaking pencil lead of specific dimensions and thickness. The lead is delicately fractured at a predetermined angle on a surface. This applied force induces slight deformations in the structure at the fracture site, leading to the transmission of acoustic waves throughout the material. Hence, the PLB tests were conducted in this study to collect AE signals. In this method, the lead is intentionally broken at a 45° angle relative to the plate’s plane. In the current study, a rectangular array consisting of four sensors (S1, S2, S3 and S4) was utilized for localization on the CFRP plate, as depicted in Figure 1. An advantageous feature of the proposed algorithm is its flexibility, as there are no restrictions on the precise placement of the sensors. The signals were preprocessed by the 2/4/6-AST preamplifier, which has a wide frequency band ranging from 50 kHz to 1 MHz, before being transmitted to the PAC Samos-48AE test apparatus (Princeton, NJ, USA), as shown in Figure 2. The AE sensor exhibited its highest sensitivity at approximately 520 kHz, achieving a maximum response of −60 dB. To eliminate noisy signals, an amplitude threshold of 45 dB was set. Other important AE parameters, including the peak definition time, hit definition time, hit lock time and maximum time duration, were selected as follows: 50 μs, 200 μs, 300 μs and 1024 μs, respectively. The sampling rate was set to 1 MHz. Due the reflection of AE signals, a single sensor could receive more than one signal during one AE source event. A comparison between the actual AE signal and its reflected wave signal reveals that the amplitude of the reflected wave was smaller. The amplitude of the original signal was greater than 85 dB. Consequently, AE signals with amplitudes below 85 dB were filtered out.

### 2.3. Quasi-Static Indentation (QSI) Test Method

**Figure 3:**
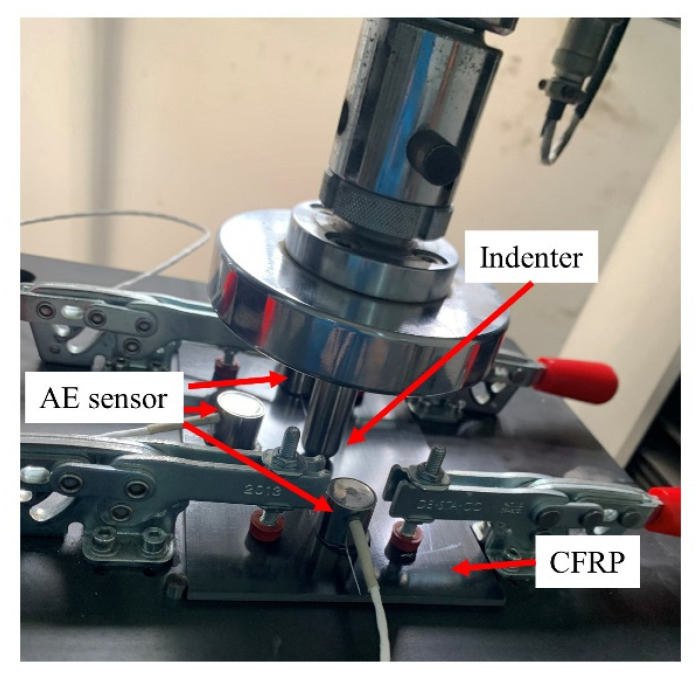
Illustration of QSI test.

The QSI tests were conducted using a hemispherical ended cylindrical impactor with a diameter of 16 mm in CMT4304 test machine produced by Meters Industrial Systems Co. (Shenzhen, China), as shown in Figure 3. The indentor rate was set as 1 mm/min. Under early-stage QSI, the indentor induced matrix cracks and delamination beneath the surface, while no significant visible damage evidence was left on the structure surface. This type of damage was referred to as barely visible indentation damage (BVID) in this study. The indentor displacement was set as 2.5 mm to induce BVID, characterized by barely visible indentation on the load surface, and the induced internal delamination. The same sensor arrangement was utilized to record the AE signal during QSI loading.

## 3. The Enhanced Delta-T Localization Method

### 3.1. Methodology

The novel localization approach used in this study introduces a new calculation parameter: characteristic vector (CV). A CV is a six-dimensional vector, where each dimension represents the difference in signal arrival time recorded by sensor pairs. The CV of a signal corresponds uniquely to its location and possesses specificity. By comparing the magnitude of the CV of a test signal to the nodes in the CV database, which is a collection of CVs at predominated nodes, the relative location relationship between the test signal and the CV database nodes can be determined. Therefore, the proposed algorithm achieves localization through two steps. Firstly, by calculating the Euclidean distance between test signal’s CV and CV database, it identifies the grid area with the average minimum Euclidean distance. Then, it utilizes the weighted centroid algorithm to make the final localization prediction. The detailed calculation process of the localization algorithm and the introduced parameters (such as CV and target search area) are further elaborated below.

Unlike most existing methods that require the interpolation fitting of the Δt curves for signal localization, this study uses the newly introduced characteristic vectors (CV) for localization. Before describing the CV, a brief introduction to the wavelet transform is given. The wavelet transform is an effective time–frequency transformation approach, providing a high level of precision in both time and frequency domains. In this study, the Gabor wavelet is adopted due to its high resolution in both the time and frequency domains. To construct the CV database, the CWT [18,20,24] of AE signal is calculated. *f*(*t*) is a function, and the *CWT* of *f*(*t*) is defined as follows:

(1) $$CWT(a,b)=\frac{1}{\sqrt{a}}\int_{-\infty }^{+\infty }f(t)\psi^{*}(\frac{t-b}{a})dt$$

where parameters *a* and *b* stand for the scale and time shift of the wavelet. *Ψ*(*·*) is the basic wavelet function. The continuous wavelet transform is reversible, which could be reconstructed using the following equation:

(2) $$f(t)=\frac{1}{C_{\psi }}\int_{a}\int_{b}CWT(a,b)\frac{1}{\sqrt{a}}\psi (\frac{t-b}{a})\frac{1}{a^{2}}dadb$$

*C_Ψ_* is a constant that depends on the wavelet function and satisfies the admissibility condition:

(3) $$C_{\psi }=\int_{-\infty }^{+\infty }\frac{|\Psi (\omega ){|}^{2}}{\left|\omega \right|}d\omega <\infty$$

where *Ψ*(*ω*) denotes the Fourier transform of wavelet used. The wavelet function must be chosen that, for any *ω*, satisfies the following expression:

(4) |Ψ(ω)|<∞

and

(5) ∫ψ(t)dt=0

According to Jeong [19], the peak of the magnitude of WT in time–frequency domain represents the arrival time of a specific frequency band component of the signal. In their PLB test, the arrival time of PLB source event is accurately determined from the peak magnitude of WT. In this research, a dynamic threshold method is employed to determine the arrival time of the chosen frequency band based on CWT. A new parameter is defined here, known as the characteristic arrival time (CAT). The CAT of the signal is defined as the time when the given frequency component of signal first crosses 90% of the maximum magnitude. It can be inferred that the difference in characteristic arrival time recorded by the sensors represents the difference in the arrival time of signals in a specific frequency band. The CAT from the signal to the sensor is represented as CAT_i_, where i denotes the label of the sensor. For the purpose of illustration, a schematic diagram of AE sensor receiving source event is presented in Figure 1. In this study, four sensors are employed that create the six-sensor pair recording the difference in CATs (ΔCAT) for the signal. The CV is a collection of ΔCATs arranged in order. Each dimension of the CV consists of the ΔCAT_i_ values calculated via a pairs of sensors. The CV is organized in the sequence of differences in time of arrival recorded via the sensor pairs S1–S2, S1–S3, S1–S4, S2–S3, S2–S4 and S3–S4. The PLB tests are repeated until all the grid nodes in the interest area have collected the available signals. In essence, the CV database is an accumulation of CVs.

Mathematically, the source event localization is an inverse problem. The establishment of the CV database has obtained a one-to-one mapping relationship between the signal positions and CVs. In order to quantitatively represent the spatial relationship between the test signal and the CV database nodes, the Euclidean distance is adopted. This distance measure is suitable for calculating the absolute distance between two points in a high-dimensional space. Thus, the Euclidean distance is employed to calculate the distance between CV of test signal and CV database grid nodes. Thus, *x*, *y* are the two *N*-dimension points *x* = (*x*^1^, *x*^2^, …, *x^N^*) and *y* = (*y*^1^, *y*^2^, …, *y^N^*). The Euclidean distance *d_xy_* is defined as follows:

(6) $$d_{xy}=\sqrt{\sum_{k=1}^{N}{\left(x^{k}-y^{k}\right)}^{2}}$$

The Euclidean distance matrix (EDM), as defined in Equation (7), is obtained by calculating the Euclidean distance between the test signal CV and each CV in the database.

(7) $$EDM=\left[\begin{array}{cccc} d_{11} & d_{12} & \ldots & d_{1N}\\ d_{21} & d_{22} & \ldots & d_{2N}\\ \vdots & \vdots & \ddots & \vdots \\ d_{N1} & d_{N2} & \ldots & d_{NN} \end{array}\right]$$

The average Euclidean distance between the CV of test signal and four nodes in each square grid of the CV database is determined via the following equation:

*d*_*aed*_ = (*d*_*i*,*j*_+ *d*_*i*,*j*+1_ + *d*_*i*+1,*j*_ + *d*_*i*+1,*j*+1_)/4 (8)

The above equation is iteratively calculated, and the target search area is defined as the grid area determined by the values of i and j when the equation obtains the minimum value. The Euclidean distance values of the four nodes within the target search area are recorded for further calculations. This step enables the initial determination of the grid location where the signal is located.

A weighted centroid localization method is then performed to estimate the precise location of the actual signal. We assume that *D*_1_, *D*_2_, *D*_3_ and *D*_4_ denote the Euclidean distances between the CV of the actual signal and the four nodes of the target location area in CV database. The weight of each node in the target search location area is calculated using the reciprocal of Euclidean distance, as shown in the following equation:

*f_i_* = 1/*D_i_* (i = 1, 2, 3, 4) (9)

where *f_i_* represents the weight of node *i* on the target grid, and *D_i_* is the Euclidean distance. In the weighted centroid method, shorter Euclidean distances are given more weight than larger Euclidean distances. One of the grid nodes in the target search area is set as the origin of the coordinate system (in this paper, the node in the lower left corner of the tested region is set as the origin). The horizontal axis represents the *x*-axis, while the vertical axis represents the *y*-axis. Assuming that the predicted location of the actual signal is (*X_p_*, *Y_p_*), the weighted centroid localization can be calculated as follows:

(10) $$X_{p}=\sum_{i=1}^{4}\left(f_{i}\times X_{i}\right)/\sum_{i=1}^{4}f_{i}$$

(11) $$Y_{p}=\sum_{i=1}^{4}\left(f_{i}\times Y_{i}\right)/\sum_{i=1}^{4}f_{i}$$

### 3.2. Localization Case Study

**Figure 4:**
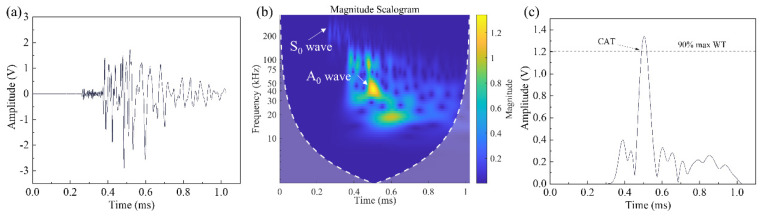
(**a**) A typical waveform of PLB AE signal, (**b**) CWT of AE signal and (**c**) magnitude of CWT at 45 kHz.

To construct the CV database, five PLB tests are conducted at each designated node to determine the average and eliminate any obvious erroneous data. Figure 4 shows a PLB test signal and corresponding CWT. The lead is broken at 45° to the plate, which results in the presence of both the S_0_ mode and A_0_ mode in the signal, as depicted in Figure 4b. One potential advantage of this method is its ability to dynamically update new areas of interest within the grid in real time. Additionally, the accuracy of the proposed localization algorithm increases with higher grid resolutions. For this study, the grid resolution was set at 10 mm.

It can be found from Figure 4b that the energy of the PLB signal is mainly concentrated at 35–50 kHz. Therefore, 45 kHz is set as the characteristic frequency band of the PLB signal. A dynamic threshold method is employed to determine the arrival time of the AE signal. The dynamic threshold is set as a magnitude of 90% of the 45 kHz AE signal component to enhance the robustness.

**Figure 5:**
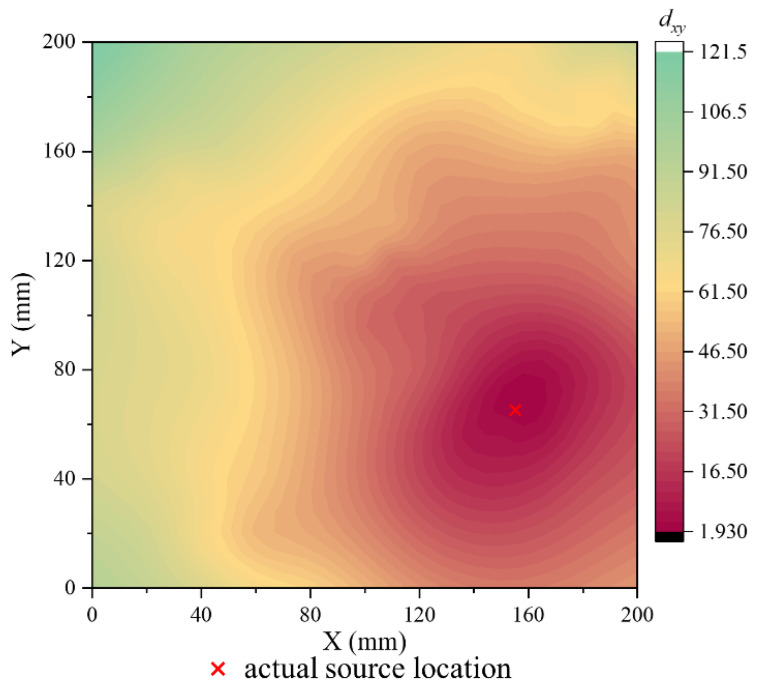
The Euclidean distance map between actual signal CV and CV database.

Figure 5 displays one of the calculation results, representing the Euclidean distance map between the CV of the actual source event and the CV database. The values of Euclidean distance range from a minimum of 1.93 to a maximum of 121.5. The red cross corresponds to the actual position of the signal occurrence. Notably, the global minimum of the Euclidean distance is observed in close proximity to the source event. Furthermore, it is evident that as the relative distance from the CV map node to the actual signal increases, the Euclidean distance increases.

**Figure 6:**
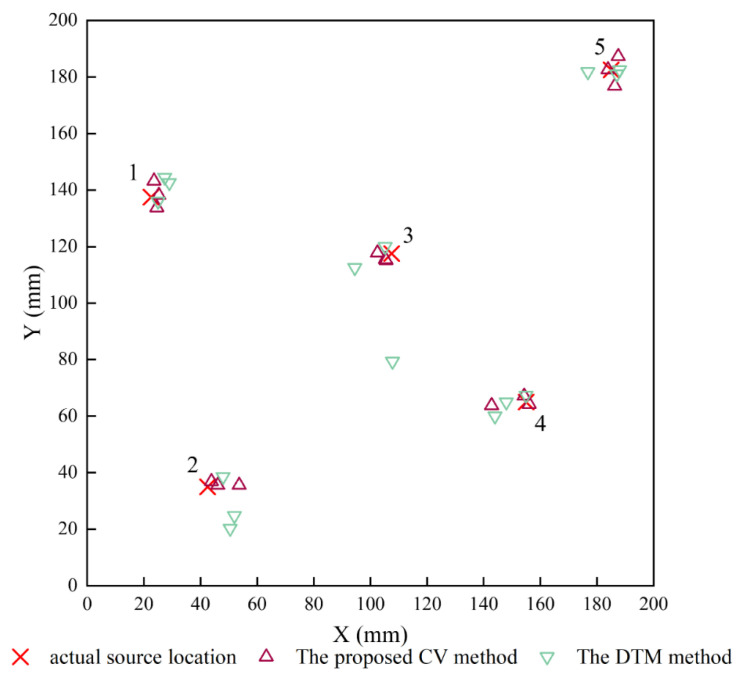
Location results using the enhanced delta T localization method.

Through an average Euclidean distance comparison, the target search area is determined. Subsequently, the weights of the four grid nodes are calculated using the reciprocal of the Euclidean distance. Finally, to locate the AE events, the weighted centroid calculation is performed through Equation (9). Figure 6 presents the experimental source location results of two localization methods alongside the actual source locations. It is evident from Figure 6 that the proposed localization method has achieved superior results. The RMS location errors depicted in Table 1 reveal that the proposed algorithm enhances the localization performance, leading to a reduction in the average location error from 3.81% to 2.31%. Further evaluation of the localization results indicates that eight out of fifteen test signals show a location error lower than 2 mm. Moreover, the proposed algorithm effectively reduces the maximum localization error to 2.87%, in contrast to the delta T mapping (DTM) method, which exhibits a maximum location error of 14.43%. There is also a significant improvement in the localization efficiency. On the premise that the CV database (Δt map) has been drawn, the traditional DTM method takes several minutes to complete a localization, whereas the proposed algorithm only requires an average of 2 s to obtain more accurate location results. More importantly, the proposed method enables batch localization. This method fulfills the real-time structural health monitoring requirements for composite materials.

## 4. Experimental Results and Discussion

### 4.1. QSI Mechanical Response

**Figure 7:**
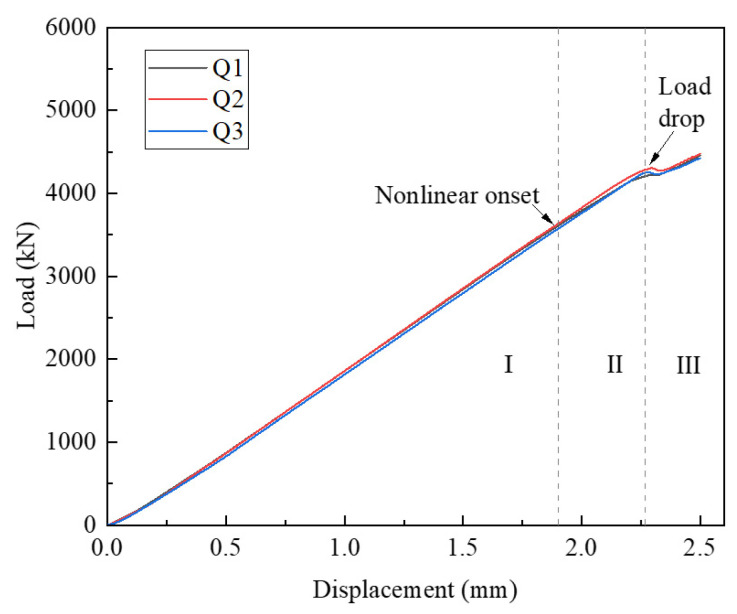
QSI load–displacement curve.

Figure 7 illustrates the load–displacement relationship of the three QSI points. It can be seen that the curves of the three load points have good consistency. Based on the response curve trend, the entire process from loading to the onset of delamination can be divided into three stages: (I) The linear relationship between load and displacement, at this stage, indicates that there is no serious damage to the internal integrity of the structure at this time. (II) The relationship between load and displacement transitions from linear to nonlinear. (III). The initial drop load is associated with the onset of delamination damage. At this stage, delamination damage begins to expand, but the loads that produce delamination initiation usually do not leave visually visible damage on the surface of the composite. Therefore, it is highly important to clarify the development and evolution of internal damage before delamination damage initiation occurred to improve the damage tolerance of composites.

### 4.2. Damage Identification Based on CWT

**Figure 8:**
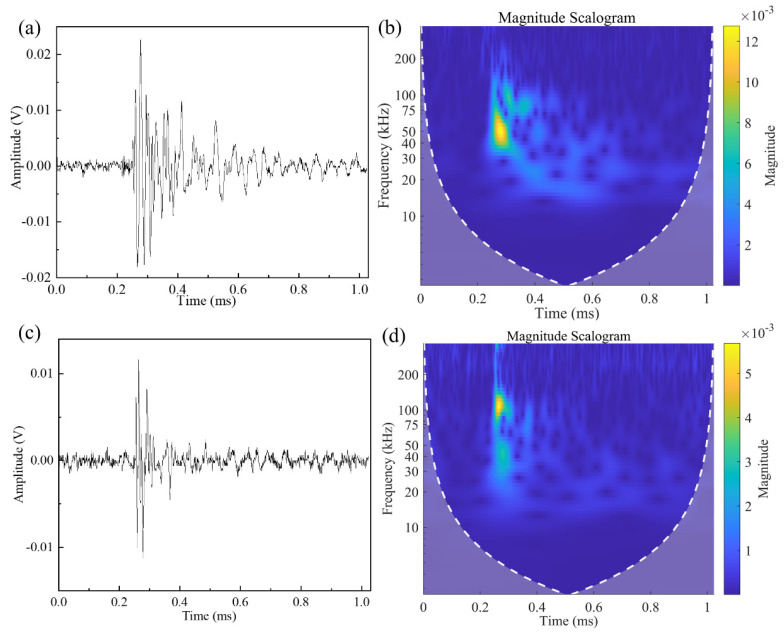
(**a**) The waveform of matrix cracking, (**b**) CWT of matrix cracking signal, (**c**) waveform of delamination and (**d**) CWT of delamination signal.

AE signals recorded during the QSI experiments, as depicted in Figure 3, are utilized for damage mode classification. Two types of signals are shown in Figure 8.

For the purpose of ensuring repeatability in classification, only AE signals recorded via sensor 1 are utilized for damage identification. Figure 8a,c depicts the AE waveforms of two types of signals, with the corresponding CWT shown in Figure 8b,d. The signal shown in Figure 8a has a longer duration time and lower main frequency band, ranging from approximately 40 kHz to 60 kHz. In contrast, the signal presented in Figure 8c has a shorter duration and a higher frequency band, ranging from 100 kHz to 150 kHz. The peak frequency of the matrix cracking signal is significantly lower than the delamination signal. The characteristics of these two types of AE signals are consistent with the matrix cracking and delamination damage mentioned in [25,26,27]. Matrix cracking and delamination are two different presentation modes of matrix damage. This can be derived from the CWTs of the two AE signals. In the CWT of matrix cracking, as shown in Figure 8b, the main signal components are distributed in the 40 kHz to 60 kHz frequency band. In Figure 8d, it can be observed that there are two distinct main frequency ranges, namely 40 kHz to 50 kHz and 100 kHz to 150 kHz. Both two types of signals have main components distributed in the 40 kHz to 50 kHz frequency band, indicating that both matrix cracking and delamination involve matrix damage.

As mentioned in the above section, the peak magnitude in the CWT represents the arrival time of the principle component of the signal. It is worth noting that both matrix cracking and delamination damage have considerable components in the 40 kHz–50 kHz band, which is similar to the PLB signal. Therefore, by selecting the appropriate frequency band, the composite matrix cracking and delamination damage can be localized using the CV map established through the PLB test.

### 4.3. Evolution of Matrix Cracking and Delamination

**Figure 9:**
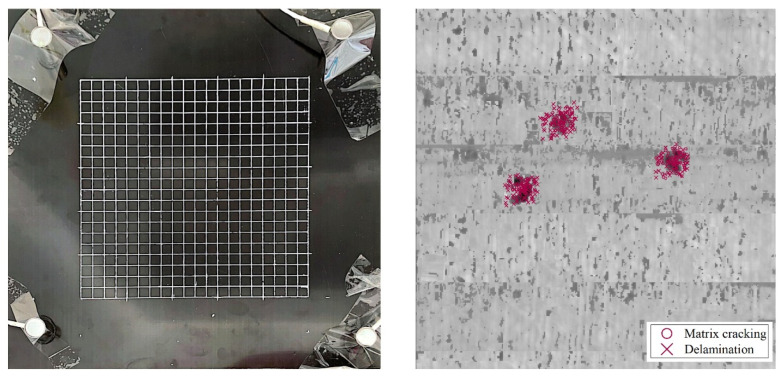
The localization results from the QSI test.

Three QSI tests are performed on a CFRP plate represented by red dots in Figure 9. Upon the visual examination of the composite plate surface, as depicted in Figure 9, it is observed that the 2.5 mm QSI loading does not leave visible damage to the plate. C-scan testing reveals internal matrix cracking and delamination damage in three indentation areas.

The predicted localization of the damage signals within the indentation area demonstrates the robustness of the localization algorithm, as shown in Figure 9, despite some errors in the signal localization. Several factors may contribute to the localization errors. Firstly, internal damage within the structure can alter the propagation path of the acoustic emission signals. Secondly, the indenter also acts as a partial conductor, resulting in different conduction characteristics compared to the composite material, which can alter the frequency components of the signals.

**Figure 10:**
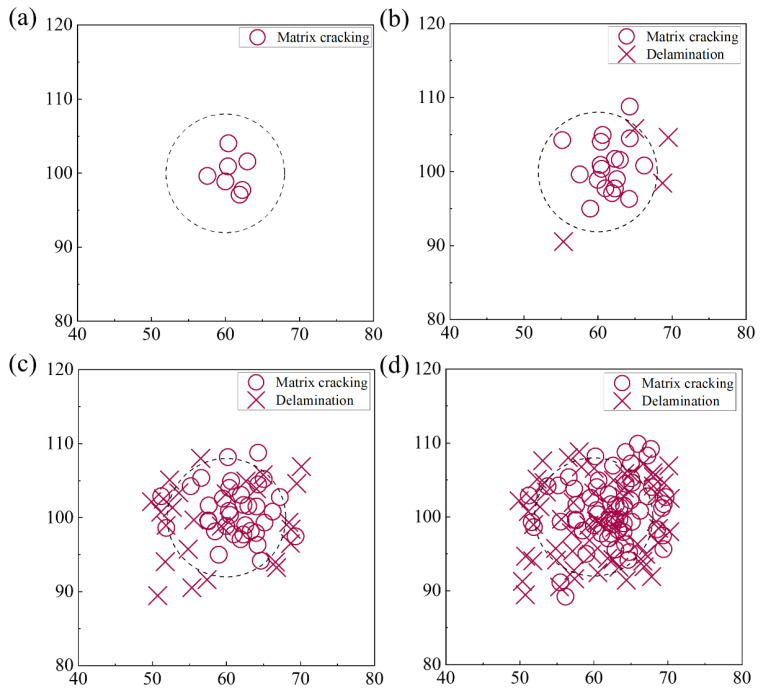
(**a**) Damage evolution in laminate CFRP under early-stage QSI: (**a**) displacement = 0.6 mm; (**b**) displacement = 1.2 mm; (**c**) displacement = 1.8 mm; (**d**) displacement = 2.4 mm.

The signal damage mode is identified through the recognition of the maximum magnitude of the CWT. The identified localized damage signals for matrix cracking and delamination are depicted in Figure 9. Both matrix cracking and delamination are primarily observed and identified in the indentation area. From the perspective of damage distribution, it can be found that the distribution of matrix cracking appears to be relatively compact, while the delamination damage is more dispersed and extends outward. In order to further illustrate the evolution process of the two types of damage under early-stage QSI loading, Figure 10 shows the damage signals recorded during the loading process.

Figure 10a–d presents the damage evolution process of laminate CFRP under QSI loading up to delamination initiation. As shown in Figure 10a, the matrix cracking initially occurs near the center of the indenter. This is mainly because the maximum deflection occurs at the center of the indenter, which causes the greatest in-face compressive stresses above the midface and the greatest tensile stresses below the midface. It can be seen that up to 1.2 mm, matrix cracking is basically distributed within the diameter of the indenter. The accumulation of matrix cracking signals at the indentation area to a certain extent indicates a localized reinforcement in matrix cracking damage. When the indenter displacement increases to 1.8 mm, the matrix cracking is extended to the outside, and it can be found that the signal of matrix cracking in the outside of the indenter region starts to increase. At this time, the matrix cracking damage in the indenter region is close to saturation, and the external matrix begins to dissipate the energy of the indenter. Based on the damage distribution at the indenter at 1.8 mm shown in Figure 10c, it can be inferred that the nonlinear phase of the mechanical response curve is associated with matrix cracking. This is because at 1.8 mm, which is also before the onset of curve nonlinearity, the matrix cracking signal has accumulated to saturation, and the mechanical properties of the structure are affected.

The delamination damage first appears at the edge of the indentation area, as shown in Figure 10b. This is because under the indentation load, the delamination damage is mainly generated through the relative slip between layers. Due to the discontinuity of the indentation load at the indenter edge, the maximum interlayer relative slip occurs at the indenter edge. In Figure 10c, it can be found that the delamination damage extends inward and outward along the radial direction after the appearance of the delamination damage signal. The amount of delamination damage grows rapidly during the 1.8 mm–2.4 mm displacement. The rapid growth results in macroscopic delamination damage initiation, leading to the impaired load carrying capacity of the specimen and dropout in the mechanical response curve.

### 4.4. Damage Energy Evolution during QSI

**Figure 11:**
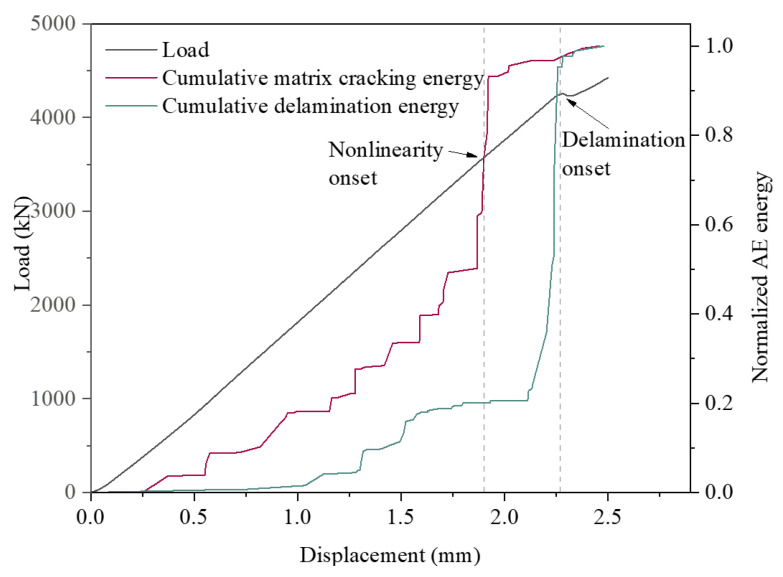
Load and cumulative AE energy versus indentation.

As shown in Figure 8, the load curves at the three indentation points are in good agreement. The load curve at Q1 is selected to analyze the energy evolution of the two damage modes up to the onset of delamination. The load curve and normalized AE energy for both damage modes versus indentation displacements are depicted in Figure 11. The characteristics of the load response curve can be found to be closely related to the energy of the matrix cracking and delamination damage modes.

It can be observed that matrix cracking is the first damage mode to occur. Prior to the nonlinear phase, the matrix cracking energy develops at an essentially steady rate of increase. When the onset of nonlinearity is reached, i.e., 1.9 mm displacement, there is a period of significant and large increase in the matrix cracking signal. The matrix cracking energy near the onset of nonlinearity accounts for about 48% of the entire pre-matrix cracking energy. This indicates that the rapid expansion of matrix cracking occurs at this stage, and more high-energy matrix cracking signals appear. The same conclusion can be obtained, according to Figure 10. Based on the damage at the indenter at 1.8 mm shown in Figure 10c, it can be inferred that the nonlinear phase of the mechanical response curve is associated with matrix cracking. This is because at 1.8 mm, which is also before the onset of curve nonlinearity, the matrix cracking signal has accumulated to a considerable extent. On the other hand, it suggests that the cause of the nonlinearity of the load curve is the rapid expansion of matrix cracking damage. The nonlinear onset occurs through the rapid extension of matrix cracking.

Based on the distribution of the normalized delamination damage energy, it can be inferred that the microscopic delamination damage signals have started to accumulate before the initiation of delamination damage. Thus, the cause of the first significant drop in the load curve is not literally the emergence of delamination damage, as the delamination signals began to appear and develop before the drop. The delamination damage initiation corresponds to the appearance and rapid development of high-energy delamination damage in a short period of time. This process occupies the stratification damage, accounting for nearly 80% of the energy of the entire pre-stratification damage mode. The delamination damage in the early stage of expansion is scattered, as shown in Figure 10b,c. Although there are more delamination damage signals at this time, they cannot be connected to form a large area of delamination damage. Therefore, the effect of the delamination damage on the trend of mechanical response curve is small. When multiple high-energy delamination damage signals appear and develop to saturation, the dispersed delamination signals connect to create macroscopic delamination damage. The emergence of macroscopic delamination leads to a reduction in the load carrying capacity of the structure and, consequently, to dropped loads.

## 5. Conclusions

In this study, the localization, classification and evolution of matrix cracking and delamination damage in composites before and after delamination initiation under low-velocity impact loading were simulated via quasi-static indentation experiments. This is of great significance for clarifying the damage extension mechanism of composites under low-velocity impact and improving the damage tolerance of composites. The main contributions of this study can be categorized into the following parts:

This study proposes a novel wavelet-based damage localization and identification in laminated CFRP plate and provides experimental validation. The algorithm addresses the limitations of existing localization method in composite material, which require multiple interpolations to fit ΔCAT curves, which occur by introducing a six-dimensional CV based on CWT. The experimental validation demonstrates that the localization of the proposed algorithm exhibits promising performance in terms of both accuracy and efficiency. Specifically, the average location error in PLB tests is reduced from 3.81% (using the DTM method) to 2.31% (using the proposed method). While the DTM method typically takes several minutes to perform location, the proposed algorithm achieves higher accuracy results within approximately 2 s.

Two damage modes, namely matrix cracking and delamination, are identified via early-stage QSI loading through the frequency band where the peak magnitude of CWT is located. Based on the frequency composition of two types of damage signals, it is inferred that matrix cracking and delamination are different presentation modes of matrix damage. The early-stage QSI results of CFRP plate indicate that there are different evolution directions for matrix cracking and delamination. Matrix cracking mainly occurs in a relatively localized area, which is a self-enhancing damage mode. Delamination damage started to initiate at the edge of indenter, and it extends simultaneously to the inside and outside of the indenter.

The trend of the mechanical response curve correlates with the development and accumulation of internal damage. The nonlinear phase of the mechanical response is associated with the appearance of a high-energy matrix cracking signal. A considerable number of delamination damage signals have already emerged before the onset of macroscopic delamination damage. Macroscopic delamination damage appears to occur after the rapid clustering of delamination damage signals.

## Figures and Tables

**Table 1:** Summary of the prediction errors in PLB tests.

Source Event Number	The Proposed Method	The DTM Method
1	4.32	11.66
2	5.74	20.9
3	3.68	28.86
4	5.28	12.12
5	4.12	2.58
Average error	4.62, 2.31%	7.61, 3.81%

Unit (mm).

## Data Availability

The data presented in this study are available on request from the corresponding author.
